# Detection, numerical simulation and approximate inversion of optoacoustic signals generated in multi-layered PVA hydrogel based tissue phantoms

**DOI:** 10.1016/j.pacs.2016.10.002

**Published:** 2016-10-21

**Authors:** E. Blumenröther, O. Melchert, M. Wollweber, B. Roth

**Affiliations:** Hannover Centre for Optical Technologies (HOT), Interdisciplinary Research Centre of the Leibniz Universität Hannover, Nienburger Str. 17, D-30167 Hannover, Germany

**Keywords:** Optoacoustics, PVA hydrogel phantom, Approximate signal inversion

## Abstract

Optoacoustic (OA) measurements can not only be used for imaging purposes but as a more general tool to “sense” physical characteristics of biological tissue, such as geometric features and intrinsic optical properties. In order to pave the way for a systematic model-guided analysis of complex objects we devised numerical simulations in accordance with the experimental measurements. We validate our computational approach with experimental results observed for layered polyvinyl alcohol hydrogel samples, using melanin as the absorbing agent. Experimentally, we characterize the acoustic signal observed by a piezoelectric detector in the acoustic far-field in backward mode and we discuss the implication of acoustic diffraction on our measurements. We further attempt an inversion of an OA signal in the far-field approximation.

## 1 Introduction

Recent progress in the field of optoacoustics (OAs) has been driven by tomography and imaging applications in the context of biomedical optics. Motivated by their immediate relevance for medical applications, high resolution scans on living tissue proved the potential of this optical absorption based measurement technique [1], [2], [3]. Requiring a multitude of detection points distributed around the source volume, OA tomography allows for the reconstruction of highly detailed images, see, e.g. Ref. [4], assuming a mathematical model that mediates the underlying diffraction transformation of OA signals [5], [6], [7], [8]. However, for most cases of *in vivo* measurements, especially on humans, it is not feasible to place ultrasound detectors at the far side of the illumination source with the “object” in between, i.e. to work in *forward mode*. Instead, it is worked in *backward mode*, where detector and irradiation source are positioned on the same side of the sample. Using elaborate setups combining the paths of light and sound waves, it is possible to co-align optical and acoustic focus within the sample, without the OA-detector blocking the optical illumination. By scanning over a multitude of detection points, it is then possible to produce 3D images [9]. E.g. Fabry–Perot polymer film based OA ultrasound mapping devices that are naturally inclined towards backward-mode measurements and proved useful to provide 3D images with high spatial resolution where reported in Ref. [10].

A conceptually different approach is to perform measurements by means of a single, unfocused transducer. Although it is not possible to obtain OA images of arbitrary 3D objects with a fixed irradiation source and a single detection point, useful information of the internal material properties can be gained. The 1D absorption profile contains information about the absorber concentration as well as its depth distribution. In this regard, acoustic near-field measurements by means of a transparent optoacoustic detector were shown to reproduce the depth profile of absorbed energy density and absorption coefficient without the need of extensive postprocessing [11], [12], [13], [14]. However, requiring close proximity (in the order of the lateral source extent) and plane wave symmetry, near-field conditions are unrealistic considering most measurement scenarios. In contrast, the acoustic far-field regime allows for a much higher experimental flexibility, albeit at the cost of the straight-forward interpretation of the measurements. More precisely, in the far-field, when the distance between detector and source is large compared to the lateral extent of the source, OA signals exhibit a diffraction-transformation, which is characteristic for the underlying system parameters [15], [16], [17], [18]. Here, aiming at the reliable measurement of material properties it is fundamental to understand the entire signal shape. In particular, in the acoustic far-field, OA signals of layered media exhibit a train of compression and rarefaction peaks and phases, signaling a sudden change of the absorptive characteristics. Regarding more complex structures intuition falls short and thus suitable simulations are needed for their interpretation. To validate the numerical approach, the comparison of the calculated signals with experimental measurements on structures with *a priori* known properties is essential. By investigating both, simulation of and measurement on a layered structure, knowledge can be obtained and subsequently transferred to more complex problems.

In the presented article, we thoroughly prepare and analyze polyvinyl alcohol hydrogel (PVA-H) phantoms, comprised of layers doped with different concentrations of melanin. The acoustic properties of the PVA-Hs match those of soft tissue, i.e. human skin [19], [20]. Note that melanin is the main endogenous absorber in the epidermis [21], and thus can be used to model melanomas. In this regard, layers with higher concentrations of melanin absorb a greater amount of photothermal energy and expand more intensely than surrounding layers with low concentrations. The stress waves emitted by these OA sources, detected in the acoustic far field after experiencing a shape transformation due to diffraction, are put under scrutiny here. Therefore, experimental measurements are complemented by custom numerical simulations. Besides analytic theory and experiment, the latter form a “third pillar” of contemporary optoacoustic studies [5].

The article is organized as follows. In Section 2 we describe the experimental setup and elaborate on the preparation of the tissue phantoms used for the measurements. In Section 3 we recapitulate the theoretical background of optoacoustic signal generation and detail our numerical approach to compute the respective signals for point-detectors, followed by details of the experimental results and complementing simulations in Section 4. We summarize our findings in Section 5.

## 2 Methods and material

In the following, the experimental setup is presented with focus on the phantom preparation process and arrangement of the layered tissue samples.

### 2.1 Photoacoustic measurement setup

For the detection of the OA pressure transient a self-built piezoelectric transducer is employed. This ultrasound detector is composed of a 9 μm thick piezoelectric polyvinylidene fluoride (PVDF) film with ∼50 nm indium tin oxide (ITO) electrodes sputtered on both sides. An in depth description of the detector is beyond the scope of this article and will be discussed elsewhere. Note that a similar acoustic detection device was employed in Ref. [12]. The active area of the detector is circular with a diameter of 1 mm. As acoustic backing layer a piece of hydrogel was prepared and placed on top of the detector with a drop of distilled water to ensure acoustic coupling. The detector can be seen as acoustically transparent, this is due to identical acoustic impedances of the backing layer and the phantom in addition to the small extent of the PVDF film in comparison to the acoustic wavelengths [11].

**Fig. 1:**
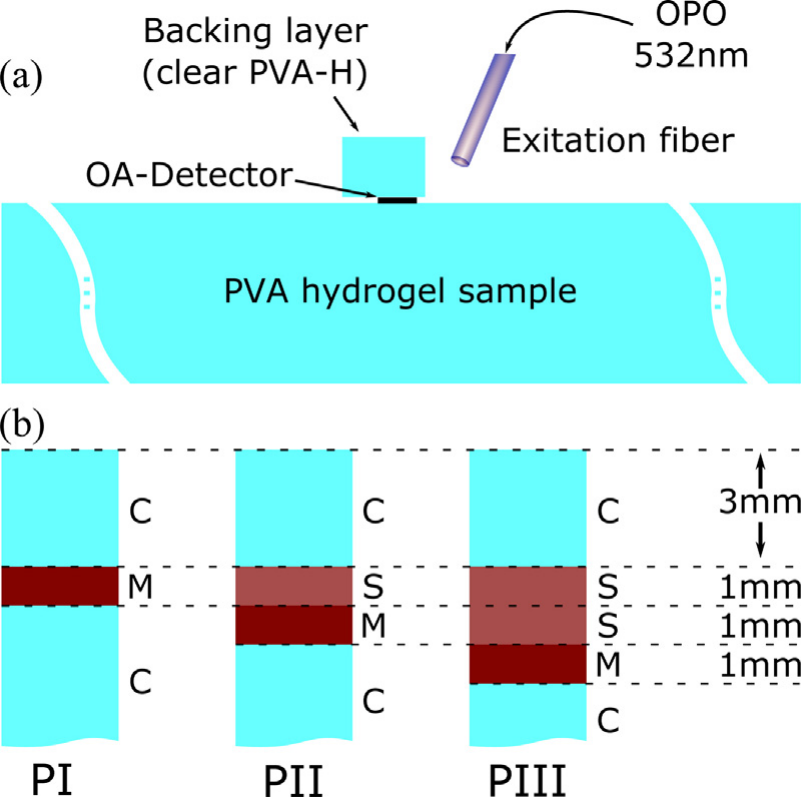
Sketch of the experimental setup. (a) Arrangement of the components as discussed in the text, see Section 2. (b) Layer composition of the three different phantoms PI, PII and PIII used for our measurements and the numerical simulations reported in Section 4. The label “C” represents clear PVA-H, “S” labels low absorption, and “M” stands for high absorption. Note that, the clear layer at the bottom is 10 mm thick. Thus, signal reflections from interfaces of materials with differing acoustic properties occur well outside the measurement range in that direction.

In contrast to the numerical approach followed in the subsequent section, the irradiation in our experimental setup was adjusted to an angle approximately 20° off the plane normal, with the light entering the phantom in close proximity of the detector, see Fig. 1. For the excitation of OA signals, an optical parametric oscillator (NL303G + PG122UV, Ekspla, Lithuania) at a wavelength of 532 nm is coupled into a 800 μm fiber (Ceramoptec, Optran WF 800/880N). The pulse duration from the pump is 3–6 ns, well complying with stress confinement. The beam profile measured after the fiber is in good agreement with a top-hat shape, which is in accordance with the irradiation source profile considered for the numerical experiments, and parameters as detailed for the numerical simulations in Section 4.

To improve the signal-to-noise ratio and match the electrical impedance, a custom build electrical pre-amplifier is connected to the detector electrodes. The voltages, corresponding to the detected pressure, are recorded at 2 GS/s (Giga sample per second) by a high-speed data acquisition card (Agilent U1065A, up to 8 GS/s). At such sampling rates, the expected ultrasound pressure profile is highly over-sampled, thus, the point-to-point noise can be smoothed out without loss of information. A conservative estimation of the fastest changing signal features yield a time window of 20 ns over which smoothing might be carried out, corresponding to 40 consecutive data points.

### 2.2 Polyvinyl alcohol based hydrogel tissue phantoms

The tissue phantoms used in our studies are composed of stacked layers of polyvinyl alcohol hydrogel (PVA-H)[22]. The incentive to utilize PVA-H is its acoustic similarity to soft tissue, i.e. human skin [19]. In contrast to liquid phantoms such as water ink solutions [13], hydrogels have the advantage of being stackable without the need of containing walls. While liquids would intermix at interfaces and thus require solid boundaries in between, hydrogels allow sharp junctions only softened by diffusion.

Here, PVA-Hs are produced by mixing polyvinyl alcohol (Sigma-Aldrich 363146, *M*_w_ 85–124 99+% hydrolyzed) with distilled water at a mixing ratio of 1:5. Using a magnetic stirrer with heating, the dispersion is kept at 94 °C for at least 40 min while the stirring bar rotates at 350 RPM, until it becomes a homogeneous solution. Crystallites produced by freezing of water in the hydrogels would yield turbidity [23]. To obtain clear PVA-H, water soluble anti freezing agents are added. Here, when the PVA is completely dissolved, ∼45 vol% of pure ethanol was incrementally added to the aqueous solution, each time waiting for the Schlieren to dissolve. Especially after adding the ethanol it is very important to keep the vessel closed whenever possible. Note, in a closed screw neck bottle the aqueous non-polymerized solution can be stored for weeks at room temperature. However, after the flask has been reheated and opened several times, ethanol evaporation inevitably causes turbidity upon freezing.

The viscous mass can be poured into any mold to obtain the desired form. Depending on the required thickness, a commercial metal distance washer or a 3D printed plastic ring of specific height was placed in between two glass plates, thus creating very flat PVA-H cylinders. Due to the much larger lateral extent of the phantoms, compared to the depth of the absorbing layers, boundary reflections do not interfere with the optoacoustic signal.

To facilitate polymerization the phantom is subjected to one *freezing and thawing cycle*. The phantom is placed in the freezer at −14 °C for 2 days. Thawing is achieved by keeping the samples at room temperature for a few minutes, afterwards the phantoms are ready to use.

The optical properties of the samples can be manipulated by inclusion of scatterers and or absorbers. In our studies, synthetic melanin (Sigma-Aldrich, M0418-100MG) was chosen as absorber to mimic human skin or even melanocytic nevi and melanoma. Due to its robustness to temperature, the finely ground melanin can be included in the beginning of the phantom creation process, at the same time with the PVA granule. As a rough estimate, it is assumed that melanomas contain as much melanin as African skin. According to [24], the melanin concentration of dark versus very fair skin differs by a factor of ten. So as to reproduce the contrast of a melanoma in Caucasian skin the following different types of PVA-H layers were created:

(i) clear PVA-H without melanin, referred to as “C”,
(ii) PVA-H with 0.1 mg/mL of melanin, referred to as “S”(Skin), and
(iii) PVA-H with 1 mg/mL of melanin, referred to as “M”(Melanoma),

Note that the melanin concentrations specified above relate to the amount of hydrogel before the addition of ethanol. By stacking these in different order, three distinct phantoms were created, see Fig. 1. While stacking the PVA-H layers in preparation for a measurement, the individual phantom layers should be kept wet by means of distilled water in order to prevent them from sticking together with one another and, most of all, themselves. Also, a proper watery film prohibits the inclusion of air in between layers. In the presented study, we considered non-scattering material only, thus we did not add any scattering supplements.

## 3 Theory and numerical implementation

We briefly recapitulate the general theory, as convenient for our approach in Section 3.1. Subsequently, in Section 3.2, we customize the general optoacoustic Poisson integral to properly represent the layered tissue phantoms and irradiation source profile used in our experiments. Finally, in Section 3.3, we emphasize some important implications of the problem-inherent symmetries on our numerical implementation.

### 3.1 General optoacoustic signal generation

In thermal confinement, i.e. considering short laser pulses with pulse duration significantly smaller than the thermal relaxation time of the underlying material [8], [26], the inhomogeneous optoacoustic wave equation relating the scalar pressure field $p(\vec{r},t)$ to a heat absorption field $H(\vec{r},t)$ reads

(1) $$[\partial_{t}^{2}-c^{2}{\vec{\nabla }}^{2}]p(\vec{r},t)=\partial_{t}\Gamma H(\vec{r},t).$$

Therein, *c* signifies the speed of sound and Γ refers to the Grüneisen parameter, an effective parameter summarizing various macroscopic material constants, describing the fraction of absorbed heat that is converted to acoustic pressure. As evident from Eq. (1), temporal changes of the local heat absorption field serve as sources for stress waves that form the optoacoustic signal. Following the common framework of stress confinement [5], we consider a product ansatz for the heating function in the form

(2) $$H(\vec{r},t)=W(\vec{r})\delta (t),$$

where $W(\vec{r})$ represents the volumetric energy density deposited in the irradiated region due to photothermal heating by a laser pulse [27], which, on the scale of typical acoustic propagation times, is assumed short enough to be represented by a delta-function. Consequently, an analytic solution for the optoacoustic pressure at the field point $\vec{r}$ can be obtained from the corresponding Greens-function in free space, yielding the optoacoustic Poisson integral [28], [29], [30]

(3) $$p(\vec{r},t)=\frac{\Gamma }{4\pi c}\partial_{t}\int_{V}\;\frac{W({\vec{r}}^{\prime })}{\left|\vec{r}-{\vec{r}}^{\prime }\right|}\delta (|\vec{r}-{\vec{r}}^{\prime }|-\mathrm{ct})\;\text{d}{\vec{r}}^{\prime },$$

where *V* denotes the “source volume” beyond which $W({\vec{r}}^{\prime })=0$ [31], and *δ*(·) limiting the integration to a time-dependent surface constraint by $|\vec{r}-{\vec{r}}^{\prime }|=\mathrm{ct}$.

### 3.2 The Poisson integral for layered media in cylindrical coordinates

As pointed out earlier, we consider non-scattering samples, composed of (possibly) multiple plane-parallel layers, stacked along the *z*-direction of an associated coordinate system. Whereas the acoustic properties are assumed to be constant within the phantom, the optical properties may change from layer to layer. Thus, the volumetric energy density naturally factors according to

(4) $$W(\vec{r})=f_{0}f(x,y)g(z),$$

wherein *f*_0_ signifies the energy fluence of the incident laser beam on the *z* = 0 surface of the absorbing material, and *f*(*x*, *y*) and *g*(*z*) specify the two-dimensional (2D) irradiation source profile and the 1D axial absorption depth profile, respectively. Bearing in mind that we consider non-scattering media, the latter follows Beer–Lambert's law, i.e.

(5) $$g(z)=\mu_{a}(z)\exp\{-\int_{0}^{z}\;\mu_{a}(z^{\prime })\;\text{d}z^{\prime }\},$$

wherein *μ*_a_(*z*) denotes the depth-dependent absorption coefficient.

**Fig. 2:**
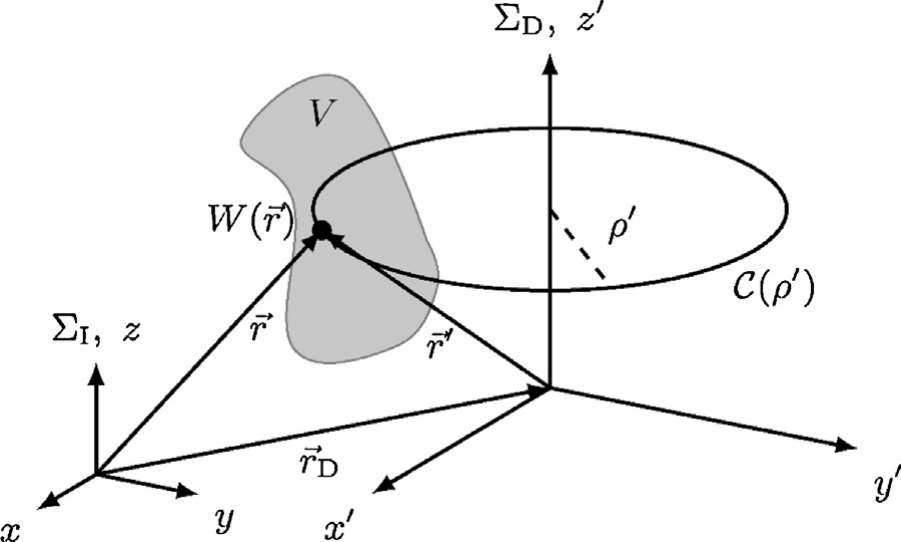
Illustration of the two reference frames, Σ_I_ with origin on the beam axis at the surface of the absorbing medium, and, Σ_D_ with origin at the detection point. Both coordinate systems are related by the transformation ${\vec{r}}^{\prime }(\rho^{\prime },\phi^{\prime },z^{\prime })=\vec{r}-{\vec{r}}_{D}$[25]. Considering cylindrical polar coordinates in Σ_D_ allows to factor the volumetric energy density $W(\vec{r})$ within the source volume *V* as detailed in the text and to pre-compute the contribution of the irradiation source profile along closed polar curves $C(\rho^{\prime })$ with radius *ρ*′. This in turn yields an efficient numerical scheme to compute the optoacoustic signal *p*_D_(*t*) at the detection point ${\vec{r}}_{D}$.

Note that, for a plane-normal irradiation with an axial symmetry, there are two useful auxiliary reference frames based on cylindrical polar coordinates: (i) Σ_I_ where $\vec{r}=\vec{r}(\rho ,\phi ,z)$ with origin on the beam axis at the surface of the absorbing medium, and (ii) Σ_D_ where ${\vec{r}}^{\prime }={\vec{r}}^{\prime }(\rho^{\prime },\phi^{\prime },z^{\prime })$ with origin at the detection point ${\vec{r}}_{D}=(x_{D},0,z_{D})$ in Σ_I_, see Fig. 2. Both reference frames are related by the point transformation ${\vec{r}}^{\prime }(\rho^{\prime },\phi^{\prime },z^{\prime })=\vec{r}-{\vec{r}}_{D}$ [25].

In Σ_I_ the irradiation source profile takes the convenient form *f*(*x*(*ρ*, *ϕ*), *y*(*ρ*, *ϕ*)) ≡ *f*_I_(*ρ*), where beam-profiling measurements for our experimental setup are consistent with a top-hat shape

(6) $$f_{I}(\rho )=\left\{\begin{array}{cc} 1, & \text{if}\;\rho \leq a\\ \exp\{-{\left(\rho -a\right)}^{2}/d^{2}\}, & \text{if}\;\rho >a \end{array}\right).$$

In Σ_D_ the constituents of the volumetric energy density read *f*_D_(*ρ*′, *ϕ*′) ≡ *f*(*x*_D_ + *ρ*′ cos(*ϕ*′), *ρ*′ sin(*ϕ*′)) and *g*_D_(*z*) ≡ *g*(*z*′ − *z*_D_), so that the optoacoustic Poisson integral, i.e. Eq. (3), takes the form

(7) $$p_{D}(t)=\frac{f_{0}\Gamma }{4\pi c}\partial_{t}\int \int_{V}\int \rho \frac{f_{D}(\rho ,\phi )g_{D}(z)}{{\left(\rho^{2}+z^{2}\right)}^{1/2}}\times \delta ({\left(\rho^{2}+z^{2}\right)}^{1/2}-\mathrm{ct})\;\text{d}\rho \;\text{d}\phi \;\text{d}z.$$

Albeit the non-canonical formulation of the Poisson integral in cylindrical polar coordinates might seem a bit counterintuitive at first, it paves the way for an efficient numerical algorithm for the calculation of optoacoustic signals for layered media.

### 3.3 Numerical experiments

#### 3.3.1 Implementation details

Considering a partitioning of the radial coordinate into *N*_*ρ*_ equal sized values Δ*ρ* = *L*_*ρ*_/*N*_*ρ*_ so that *ρ*_*i*_ = *i*Δ*ρ* with *i* = 0 … *N*_*ρ*_ − 1, the preceding factorization of the volumetric energy density $W(\vec{r})$ in Σ_D_ allows to pre-compute the contribution of the irradiation source profile in Eq. (7) along closed polar curves $C(\rho_{i})$ with radius *ρ*_*i*_ according to

(8) $$F_{D}(\rho_{i})=\lim_{N_{\phi }\to \infty }\rho_{i}\sum_{j=0}^{N_{\phi }-1}f_{D}(\rho_{i},\phi_{j})\Delta \phi ,$$

where Δ*ϕ* = 2*π*/*N*_*ϕ*_ and *ϕ*_*j*_ = *j*Δ*ϕ* with *j* = 0 … *N*_*ϕ*_ − 1, thus completing the integration over the azimuthal angle and providing the results in a tabulated manner with time complexity *O*(*N*_*ρ*_*N*_*ϕ*_). This in turn yields an efficient numerical scheme to compute the optoacoustic signal *p*_D_(*t*) at the detection point ${\vec{r}}_{D}$ since the pending integrations can be carried out with time complexity *O*(*N*_*ρ*_*N*_*z*_), in a discretized setting with Δ*z* = *L*_*z*_/*N*_*z*_ so that *z*_*k*_ = *k*Δ*z* for *k* = 0 … *N*_*z*_ − 1. Consequently, interpreting the *δ*-distribution in Eq. (7) as an indicator function that bins the values of the integrand according to the propagation time of the associated stress waves, the overall algorithm completes in time *O*(*N*_*ρ*_*N*_*ϕ*_ + *N*_*ρ*_*N*_*z*_). Note that for the special case *x*_D_ = *y*_D_ = 0, i.e. for detection points on the beam axis, Eq. (8) further simplifies to *F*_D_(*ρ*_*i*_) = 2*πρ*_*i*_*f*_I_(*ρ*_*i*_), reducing the time complexity to only *O*(*N*_*ρ*_*N*_*z*_) [32]. During our numerical simulations,^1^ for practical purposes and since we are only interested in the general shape of the optoacoustic signal in order to compare them to the transducer response, we set the value of the constants in Eq. (7) to *f*_0_Γ/*c* ≡ 4*π*. Thus, the resulting signal is obtained in arbitrary units, subsequently abbreviated as [a.u.], making it necessary to adjust the amplitude of the signal if we intend to compare the results to actual measurements. Further, to mimic the finite thickness Δw of the transducer foil, see Section 2, we averaged the optoacoustic signal at the detection point over a time interval Δt=Δw/c.

#### 3.3.2 Exemplary optoacoustic signals

**Fig. 3:**
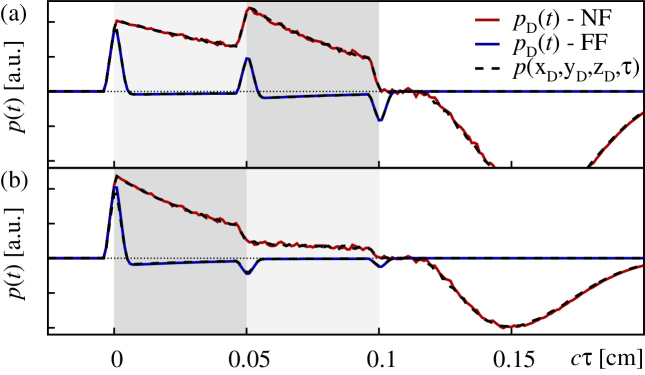
Comparison of different solvers for the optoacoustic problem for layered media in two different layer scenarios. The data curves labeled by *p*_D_(*t*) refer to an implementation in cylindrical polar coordinates according to Eq. (7). The curves are computed for a field point in the acoustic near-field (NF; red line) and far-field (FF; blue line) at *z*_D_ = −0.04 cm and *z*_D_ = −4.0 cm on the beam axis, respectively. The corresponding numerical results obtained using an implementation of Eq. (3) in Cartesian coordinates are labeled by $p({\vec{r}}_{D},\tau )$ (black dashed lines). (a) Setup where the source-volume contains two absorbing layers consisting of *μ*_*a*_ = 10 cm^−1^ in the range *z* = 0.0–0.05 cm (light-gray shaded region) followed by *μ*_*a*_ = 20 cm^−1^ in the range *z* = 0.05–0.1 cm (gray shaded region), and, (b) setup where the order of the layers is reversed.

So as to facilitate intuition and to display the equivalence of the numerical schemes implemented according to Eqs. (3), (7) in both, the acoustic near field (NF) and far field (FF), we illustrate typical optoacoustic signals in Fig. 3. Therein, the “Cartesian” solver (not detailed further) was based on a voxelized cubic representation of the source volume with side-lengths (*L*_*x*_, *L*_*y*_, *L*_*z*_) = (0.6, 0.6, 0.15) [cm] using (*N*_*x*_, *N*_*y*_, *N*_*z*_) = (1500, 1500, 150) meshpoints, whereas the solver based on cylindrical coordinates used a decomposition of the computational domain into (*L*_*ρ*_, *L*_*z*_) = (0.3, 0.15) cm and (*N*_*ρ*_, *N*_*ϕ*_, *N*_*z*_) = (6000, 360, 150). The parameters defining the irradiation source profile were set to *a* = 0.15 cm and *d* = *a*/4. As finite thickness of the transducer foil we considered a slightly oversized Δw=50μm for our numerical experiments (note that the “real” transducer foil used in our experiments has Δw=10μm).

The dimensionless diffraction parameter $D=2|z_{D}|/(\mu a_{0}^{2})$ [13], [15] can be used to distinguish the acoustic near field (NF) at *D* < 1 and far field (FF) at *D* > 1. Here, we consider the effective parameters *μ* = 〈*μ*_a_(*z*)〉 and *a*_0_ = 1.25 · *a* in case of multi-layered tissue phantoms. The simulations were performed at detection points on the beam axis, realizing NF conditions with *D* ≈ 0.15 at *z*_D_ = −0.04 cm and FF conditions with *D* ≈ 15.0 at *z*_D_ = −4.0 cm. As evident from Fig. 3, the optoacoustic NF signals are characterized by an extended compression phase in the range *cτ* = 0.0 − 0.1 cm, resulting from the plane-wave part of the propagating stress wave, which accurately trace the profile of the volumetric energy density along the beam axis, followed by a pronounced diffraction valley for *cτ* > 0.11 cm. The particular shape of the latter is characteristic for the top-hat irradiation source profile used for the numeric experiments. In contrast to this, as can be seen from Fig. 3, the FF signal features a succession of compression and rarefaction phases. Therein a sudden increase (decrease) of the absorption coefficient is signaled by a compression peak (rarefaction dip), cf. the sequence of peaks and dips at the points *cτ* = 0, 0.05, 0.10 cm in Fig. 3(a) and (b), corresponding to the boundaries of the differently absorbing layers. Further, the diffraction valley has caught up, forming rather shallow rarefaction phases in between the peaks and dips [34]. Finally, note the excellent agreement of the signals obtained by the two independent OA forward solvers.

**Fig. 4:**
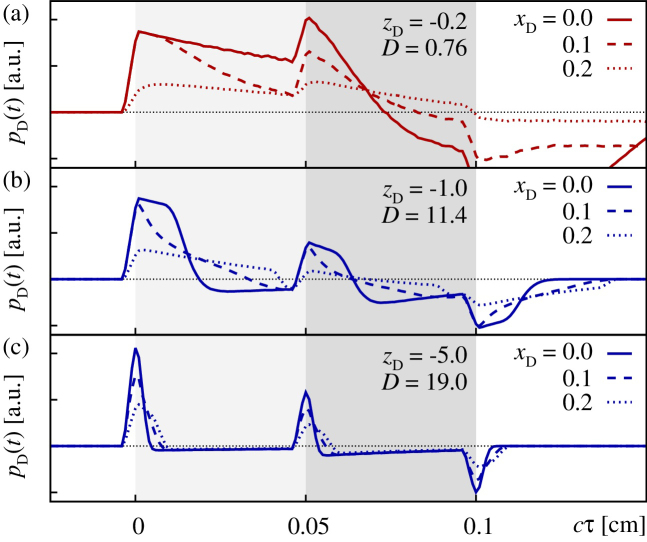
Sensitivity of the optoacoustic signal *p*_D_(*t*) on a radial deviation of the detection point ${\vec{r}}_{D}$ from the beam axis, realized by setting *x*_D_ ≠ 0 cm, as explained in the text. The subfigures refer to different distances *z*_D_, where (a) *z*_D_ = −0.2 cm is located in the acoustic NF with *D* = 0.76, (b) *z*_D_ = −1.0 cm (*D* = 11.4) in the “early” FF, and, (c) *z*_D_ = −5.0 cm (*D* = 19.0) in the “deep” FF.

In a second series of simulations we clarified the influence of the radial deviation of the detection point ${\vec{r}}_{D}$ from the beam axis. Therefore we computed the excess pressure *p*_D_(*t*) at different positions *x*_D_ ≠ 0 perceived in Σ_I_. The results for *x*_D_ = 0.1 cm, i.e. 2/3 along the flat-top part of the top-hat profile, and *x*_D_ = 0.2 cm, i.e. slightly above the 1/*e*-width of the beam intensity profile, are illustrated in Fig. 4. As evident from Fig. 4(a), for *z*_D_ = −0.2 cm, realizing a location with *D* = 0.76 in the acoustic NF, the optoacoustic signal appears to be quite sensitive to the precise choice of *x*_D_. I.e., as soon as the border of the plane-wave part of the signal is approached, the transformation of the signal due to diffraction is strongly visible. Comparing the points *z*_D_ = −1.0 cm (*D* = 11.4) in the “early” FF and *z*_D_ = −5.0 cm (*D* = 19.0) in the “deep” FF, it is apparent that the optoacoustic signal in the FF is less influenced by the off-axis deviation of the detection point, see Fig. 4(b) and (c). Also, note that with increasing distance |*z*_D_|, the interjacent rarefaction phases level off and move closer to the leading compression peaks [34]. From the above we conclude that, if we compare experimental measurements recorded in the FF with numerical simulations, we should find a good agreement between detected and calculated signals even though both exhibit different degrees of deviation from the beam axis.

#### 3.3.3 Validity and limits of the presented approach

The presented numerical approach is valid in case of homogeneous acoustic material properties. While this is an assumption that applies for the PVA-H based tissue phantoms described in Section 2, this might not sufficiently describe biological samples that are put under scrutiny in applied optoacoustic tomography. Regarding the computation of OA signals in the direct direction considering spatially heterogeneous acoustic properties, one might choose from a variety of suitable approaches, as, e.g. the Lattice–Boltzmann method [35], finite-difference method [36] or a generalized form of the Poisson integral equation [37], presented earlier as Eq. (3). A more intricate endeavor is the inversion of OA signals to initial acoustic stress fields $W(\vec{r})$ while accounting for heterogeneous acoustic properties [7]. In principle, the spatially varying acoustic velocity field $c(\vec{r})$ needs to be known before an inversion to $W(\vec{r})$. A common technique to recover $W(\vec{r})$ for a known velocity field $c(\vec{r})$ is time-reversal, i.e. solution of the 3D wave equation backwards in time [38], [39]. However, in the unfavorable case where the acoustic velocity field is not known, one might rely on a fitting parameterization by use of prior information on the acoustic properties within the computational region of interest in order to allow for a simultaneous reconstruction of $c(\vec{r})$ and $W(\vec{r})$ as demonstrated by Ref. [37]. Finally, note that, due to the already good agreement of simulated data and measurement, attenuation of ultrasound has been neglected. However, in case of homogeneous acoustic properties and known material properties, computed OA signals might be corrected for effects of acoustic dispersion in Fourier space [40], [41]. As reported in Ref. [40], this might be used vice versa to correct measured data prior to inversion via methods that do not account for acoustic attenuation. To the best of the author's knowledge, it seems that the influence of attenuation in presence of inhomogeneous acoustic material properties has not been sufficiently studied and reported in the literature, yet.

This completes the discussion of optoacoustic signals and their generation from the point of view of computational theoretical physics. Details regarding the measurements on layered tissue phantoms and custom simulations geared towards those experiments are given in the subsequent section.

## 4 Results

Below we compare measured OA signals, obtained from measurements on the three tissue phantoms PI–PIII, discussed in Section 2 and illustrated in Fig. 1(b), with custom simulations created in terms of the numerical framework detailed in Section 3. As evident from the comparison of the experimental setup with the simulation framework, there are three differences between experiment and theory which have to be kept in mind while interpreting the results. The first two points refer to the location and orientation of the irradiated absorber volume relative to the OA detector and its principal axis: (i) while the irradiation is assumed to be plain normal incident for our simulations, the direction of incidence in the experimental setup is off the plane normal by a nonzero angle. Additionally, due to unavoidable refraction at the phantom surface, the beam profile is likely to be slightly non-symmetric and divergent. Hence, the top-hat beam shape assumed in our simulation approach can only been seen as an approximation of the experimental conditions. (ii) Although it is probable that all the acoustic measurements are performed, at least to some extent, off-axis, we opt for modeling and numerical simulations in an on-axis approach. As demonstrated in Section 3.3 and illustrated in Fig. 4(c), we expect the principal signal shape in the acoustic far-field to change only at a small rate upon deviation from the beam axis. (iii) As pointed out in Section 2, the active area of the transducer has a radius of 0.5 mm, while in our simulations we compute optoacoustic signals for a point-like detector. However, upon approaching the far-field limit one expects the former intrinsic length scale not to be of significance. The apparent qualitative agreement of simulation and experiment detailed in the remainder is impressive and should suffice to validate our approach.

### 4.1 Comparison of optoacoustic signals obtained from theory and experiment

**Fig. 5:**
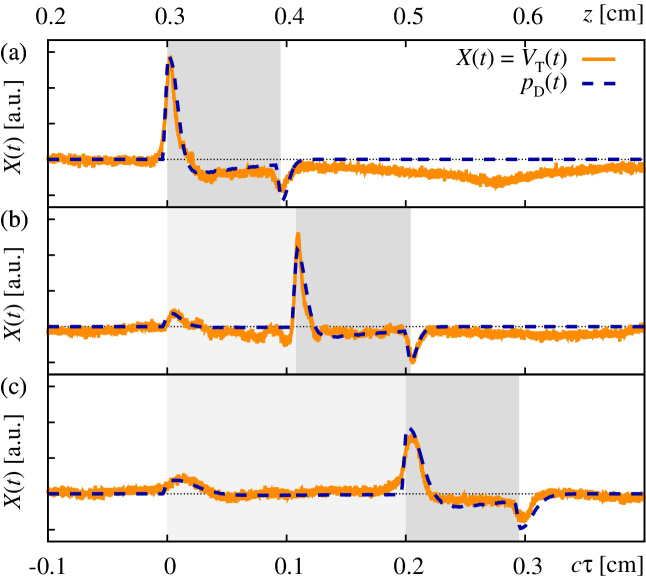
Comparison of optoacoustic signals for layered media obtained from measurements (labeled “*V*_T_(*t*)”; orange solid lines) and numerical simulations (labeled “*p*_D_(*t*)”; blue dashed lines). The top and bottom abscissas refer to the distance *z* traveled by the signal and the retarded signal depth *cτ* = *c t* + *z*_D_. The detector is located at *z*_D_ = −0.3 cm, hence the first compression peak is expected at *cτ* = 0 cm. (a) Single-layer tissue phantom PI, (b) double-layer tissue phantom PII, and, (c) double-layer tissue phantom PIII, see Fig. 1(b). Regions in different shades of gray indicate layers with different melanin concentration (cf. Fig. 1(b)). Note, the presented data was obtained in single measurement and was neither smoothed nor averaged.

The measured optoacoustic signals for the tissue phantoms PI–PIII along with the simulated curves are illustrated in Fig. 5(a)–(c). In principle all three measured curves exhibit the characteristic features expected for signals observed in the acoustic far-field. Thus these measurements are well suited for the purpose of optoacoustic depth profiling [13]. In particular, for the simulation of PI, we considered a single layer with an assumed absorption coefficient *μ*_*a*_ = 11 cm^−1^ in the range *z* = 0.3–0.395 cm (*z* is measured with respect to the origin of Σ_D_), indicated by a gray shaded region representing a highly absorbing layer (introduced as “M” in Section 2). The deviation from a layer thickness of 1 mm in the simulations accounts for small variations in the actual layer thickness of the tissue phantom as well as a putative inter-layer variation of the sound velocity. Experimentally, the detector response is measured vs. time. Therefore, any comparison between simulation and experiment is affected by the speed of sound, which, in the theoretical framework is assumed to be homogeneous throughout the source volume. The top-hat beam shape parameters within the simulation where set to *a* = 0.054 cm and *R* ≡ *d*/*a* = 1.5. Note that due to the aforementioned challenges concerning the preparation of the shape and position of the beam profile at the absorbing layer, it is merely possible to set realistic parameter-boundaries from which to choose precise values for *a* and *R*. As a remedy, we here fixed the parameter values so that they reproduce the principal features of the signal, i.e. the initial compression peak, the trailing rarefaction dip as well as the intermediate rarefaction phase, well in theory and experiment. The subsequent long and shallow rarefaction phase for *z* > 0.5 cm in Fig. 5(a) is located outside the targeted measurement range corresponding to the prepared source volume and is likely caused by acoustic reflections from the lateral boundaries of the backing layer.

Phantom PII was modeled by considering a first layer with a comparatively low absorption coefficient *μ*_*a*_ = 1.4 cm^−1^, i.e. type-S, in the range *z* = 0.3–0.408 cm (light-gray shaded region), followed by a type-M layer with *μ*_*a*_ = 11 cm^−1^ in the range *z* = 0.408–0.504 cm (gray shaded region). Therein, the beam shape parameters where set to *a* = 0.056 cm and *R* = 1.2. Here, all three expected characteristic signal features, i.e. the initial small compression peak, the interjacent high compression peak as well as the trailing rarefaction dip match well for theory and experiment.

Finally, phantom PIII was modeled by considering a type-S layer with *μ*_*a*_ = 1.4 cm^−1^ in the range *z* = 0.3–0.5 cm (light-gray shaded region) followed by a type-M layer with *μ*_*a*_ = 11 cm^−1^ in the range *z* = 0.5–0.595 cm (gray shaded region). Therein, the beam shape parameters where set to *a* = 0.08 cm and *R* = 1.2. Again, all three characteristic signal features are reproduced well by theory and experiment.

As pointed out in Section 3.3, it is necessary to adjust the scale of the amplitude of the computed OA signal if we intend to compare it to the transducer response. The respective scaling factor was obtained from the simulated and measured curves for tissue phantom PI and the same scaling factor was subsequently used in the other two cases to achieve the excellent agreement displayed in Fig. 5(a)–(c).

### 4.2 Reconstruction of the initial volumetric energy distribution

**Fig. 6:**
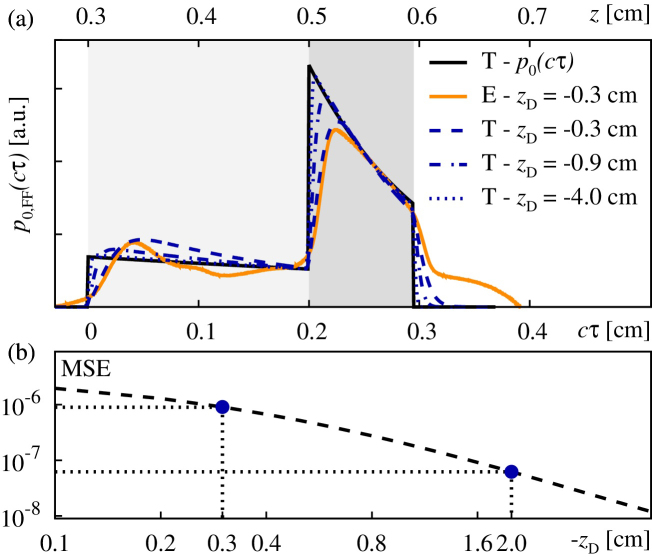
Reconstruction of the initial volumetric energy density in the far-field (FF) approximation for tissue phantom PIII, see Fig. 5(c). (a) Comparison of the exact initial distribution of acoustic stress *p*_0_ (black solid line; labeled “T”) to FF reconstructed predictors *p*_0,FF_ simulated at different measurement points *z*_D_ (blue lines; labeled “T”) and the FF reconstructed predictor derived from our measurement (orange solid line; labeled “E”). (b) Mean square error MSE between the exact initial volumetric energy density and the FF reconstructed value from the optoacoustic signals calculated at different detection points at *z*_D_ = −0.1 through −4 cm.

Owing to its immediate relevance for medical applications [2], [3], recent progress in the field of OA has been driven by photoacoustic tomography (PAT) and imaging applications [5]. Therein, aim is to reconstruct the initial acoustic stress profile to facilitate a reconstruction of the OA properties of the underlying sample via inversion. Usually, the PAT inversion input consists of a large number of OA signals, recorded on a surface enclosing the OA source volume. In contrast, considering “single-shot” FF measurements as above, the observed signals can not only be used for OA depth profiling, they can also be related to the initial volumetric energy distribution by means of a temporal derivative [13], [14], [15]. As discussed in Ref. [13], this offers the possibility to reconstruct the initial acoustic stress distribution *p*_0_(*z*) = Γ*W*(*z*) in the limit *D* ≫ 1. Note that Refs. [13], [14] used the integral of the measured acoustic signals as a visual aid for imaging purposes, cf. Fig. 9(c) of Ref. [13], and Fig. 8(b) of Ref. [14], they did not elaborate on this issue any further. Here, we attempt to explore the use of the idea above in order to obtain a predictor *p*_0,FF_ ≈ *p*_0_ in terms of a FF approximation for tissue phantom PIII. This is illustrated in Fig. 6(a), where we show the exact initial distribution of acoustic stress *p*_0_ (solid black line) by means of which the numerical simulations were carried out, together with the FF reconstructed predictors *p*_0,FF_ simulated at three different measurement points *z*_D_ = −0.3, −0.9, −4.0 cm in the acoustic FF and the FF reconstructed predictor derived from the experimental measurement. While the measurement based and simulation based predictors at *z*_D_ = −0.3 cm agree well it can be seen that, even though the simulations are carried out in acoustic FF, they still differ noticeably from the exact curve. As one might intuitively expect, an increasing distance |*z*_D_| yields a more consistent estimate. In the limit |*z*_D_| → ∞ this is limited only by the temporal averaging of the signal, implemented to mimic a finite thickness of the transducer foil.

This can be assessed on a quantitative basis by monitoring the mean squared error $\mathrm{MSE}=\sum_{i=0}^{N_{z}-1}{\left[p_{0}(z_{i})-p_{\text{0,FF}}(z_{i})\right]}^{2}/N_{z}$ in a discretized setting, with *z*_*i*_ as in Section 3.3, see Fig. 6(b). Note that in advance, the above signals are normalized in order to ensure ∑_*i*_*X*(*z*_*i*_) = 1 for both, *X* = *p*_0_ and *p*_0,FF_. As evident from the figure, the MSE might be reduced by a solid order of magnitude upon moving the signal detection from *z*_D_ = −0.3 cm to −2.0 cm and thus further into the far-field (indicated by the dashed lines in the figure).

## 5 Summary and conclusions

In the presented article we discussed an efficient numerical procedure for the calculation of optoacoustic signals in layered media, based on a numerical integration of the optoacoustic Poisson integral in cylindrical polar coordinates, in combination with experimental measurements on PVA based hydrogel tissue phantoms. In summary, we observed that far-field measurements on tissue phantoms composed of layers with different concentrations of melanin are in striking agreement with custom numerical simulations and exhibit all the characteristic features that allow for optoacoustic depth profiling. Further, in our experiments, the signal to noise ratio of single measurements was sufficiently high to omit any signal post-processing. In contrast to the experimental measurements, the simulations are performed with on axis illumination and assuming an ideal point-like detector. Nonetheless, simulation and experiment agree very well over all, which highlights the robustness of the signal analysis and simulation against small deviations. Finally, we showcased the possibility to reconstruct the initial pressure profile in a far-field approximation by numerical integration. Even though exact reconstruction would require an ideal detector in addition to an infinite distance between source and detector, the pressure profile reconstructed here (with finite distance |*z*_D_| = 1 cm and finite detector radius 0.5 mm) reproduces the initial pressure profile exceedingly knorke. In this regard, from the point of view of computational theoretical physics, it is also tempting to explore further, conceptually different signal inversion approaches, that might facilitate a reconstruction of “internal” optoacoustic material properties based on the measurement of “external” OA signals. Such investigations are currently in progress.

## Conflict of interest statement

The authors declare that there are no conflicts of interest.

## Footnotes

1 A Python implementation of our code for the solution of the photoacoustic Poisson equation in cylindrical polar coordinates, i.e. Eq. (7), can be found at [33].
